# Tracheoesophageal Iatrogenic Fistulas in ICU: Still a Pandora's Box?

**DOI:** 10.2478/jccm-2024-0044

**Published:** 2024-10-31

**Authors:** Radu Stoica

**Affiliations:** Clinical Hospital Sanador Bucharest, Romania

Tracheoesophageal fistulas (TEF) in the ICU are still considered a relatively rare, but life-threatening complication of prolonged intubation, with an incidence of approximately 0.5% of cases [1]. Classically, their occurrence was considered the result of the direct interaction between two mechanical factors: the endotracheal tube (ETT) or the tracheostomy tube placement on the membranous wall of the trachea, and the esophageal feeding tube that affects esophageal mucosa, leading to ischemic lesions and decubitus injury. The question that arises is why, despite this simple explanation, the incidence of TEF remains low? In reality, the occurrence of TEF in ICU is related to the complex interactions between patients' comorbidities and the particularities of pathophysiology and management in critically ill patients, leading to local tissue metabolic disorders and favoring fistulas' occurrence. Malnutrition, diabetes, chronic anemia, reflux esophagitis, prolonged inflammation, sepsis, hemodynamic instability, prolonged hypoxemia, vasoactive drugs or corticosteroids are the mainly factors favoring fistulas' development.

Tracheoesophageal fistulas are a challenge for intensivists, many patients require prolonged hospitalization with limited therapeutic options and conservative treatment. The prognosis is reserved and is related to the severe initial pathology, comorbidities and complications arising during the prolonged ventilation and the stay in the ICU. An important issue is related to the late diagnosis of the lesion, especially in the small or incipient fistulas, when the well-known signs (appearance of air-leak during mechanical ventilation, abdominal distension due to ventilation through the fistula opening or gastric reflux in the airways) are absent. In this case, the intensivist should be aware that indirect and non-specific signs (pulmonary recurrent infections, ARDS, difficult ventilatory withdrawal, abdominal distension) could be consider early symptoms of TEF. Despite all of “warning sings”, sometimes, this type of fistula is recognized late, when complications occurred and it is usually 4–5 cm large, and often with already epithelialized edges which makes spontaneous closure impossible.

The management of TEF is different, related to the “age” and the length of parietal defect. The conservative approach is represented by the prevention of respiratory infection, the gastro- or jejunostomy catheter placement and the general supportive treatment. Esophageal and/or tracheal stents placement is attempted in certain selected cases. For patients with a favorable general evolution, surgical treatment is the only optimal approach ensuring the physiological reestablishing of respiratory and digestive functions. The timing for surgery is sometimes difficult, as there is a dilemma between rushing the surgical intervention to prevent secondary complications, especially respiratory ones, and the postponement dictated especially by nutritional, neurological, cardio-vascular recovery or the treatment of infections. Ideally, surgery should be performed in patients with spontaneous breathing but there are situations when this was performed in patients who are still mechanically ventilated, having the indication, and the hope(!) that this will help the ventilatory withdrawal [3]. Once the surgical indication is established, the coordination between the surgical and anesthetic teams is extremely important [4].

The amplitude of the lesions is assessed by bronchos-copy, upper digestive endoscopy, and three-dimensional reconstruction computer tomography. Anesthesia is challenging due to the interference of surgery technique with the airways management. The general anesthesia is usually intravenous, avoiding losses of volatile anesthetics in the operating room during ETT change maneuvers and allowing alternative ventilation techniques (e.g., High Jet Frequency Ventilation -HFVJ). Initiation of ventilation is performed on the tracheostomy tube. An ETT is inserted above the tracheostomy tube in “pending position”.

Extubation is recommended to be performed in the operating room, which is possible in most cases if the surgical indication was properly established. If the ventilator weaning process is difficult or the resection was very extensive in a fragile tissue, the tracheostomy, a Montgomery or a Hebeler Safe-T-Tube for mechanically ventilated patients can be maintained [3]. This complex intervention requires a multidisciplinary medical team, a specific medical equipment, a dedicated service with very well-trained personnel. The postoperative outcomes are various, mainly due to the small series of patients, therefore it is not surprising that the rate of anastomotic complications are between 10% and 50% and the postoperative mortality up to 10%. The outcomes are influenced by the patient's selection and the experience of each center. A study published by Grillo (2004) on 38 patients, 27 presented post-intubation fistulas, with a mortality of 1 in 27. Postoperative vocal cord dysfunction occurred in one case, fistula recurrence after surgery was between 3 to 8.3% [5].

In conclusion, despite the advances of medicine, critically ill patients are prone to develop TEF, due both mechanical and biological favouring factors as inflammation, sepsis, metabolic disturbances, malnutrition, hypoxemia, hemodynamic instability, medication or prolonged ventilation. The intensivist should be aware about TEF occurrence in patients with recurrent pulmonary infections, ARDS from an unknown cause or difficult mechanical ventilation weaning.

Finally, I would emphasize two aspects. Few pathological conditions, once operated on, can progress from “a miserable condition”, to a maximum respiratory and dietary discomfort, and dependence on permanent medical care, to finally reach a normal life. Patients with sudden acute diseases (e.g., stroke, polytrauma) with good chances of recovery in the ICU, that develop TEF during prolonged ventilation are the ones who benefit the most.

The transfer to a service with experience in TEF surgical management should not be delayed, as recurrent complications can appear at any time.

## References

[1] Mathisen DJ, Grillo HC, Wain JC, Hilgenberg AD (1991). Management of acquired nonmalignant tracheoesophageal fistula. Ann Thorac Surg.

[2] Dartevelle P, Macchiarini P (1996). Management of acquired tracheoesophageal fistula. Chest Surg Clin N Am.

[3] Fiala P, Cernohorsky C, Cermak J, Patek J, Krepela E, Mouckova M (2004). Tracheal stenosis complicated with tracheoesophageal fistula. Eur J Cardiothorac Surg.

[4] Stoica R, Cordos I (2020). Tracheal and Bronchial Surgery: HJFV Cap 23: 343–360, in Anesthesia in Thoracic Surgery, Changes in Paradigms.

[5] Grillo HC (2011). Surgery of the tracheal and bronchi.

